# Mucobilia: An Unusual Cause of Persistent Jaundice

**DOI:** 10.7759/cureus.4565

**Published:** 2019-04-30

**Authors:** Jinendra Satiya, Tolga Erim, Alexander Restrepo, Conrad Simpfendorfer, Albert J Parlade

**Affiliations:** 1 Internal Medicine, University of Miami, John F Kennedy Medical Center, Atlantis, USA; 2 Gastroenterology and Hepatology, Cleveland Clinic Florida, Weston, USA; 3 Gastroenterology and Hepatology, West Palm Beach Veterans Affairs Medical Center, West Palm Beach, USA; 4 Surgery, Cleveland Clinic Florida, Weston, USA; 5 Radiology, Cleveland Clinic Florida, Weston, USA

**Keywords:** mucobilia, obstructive jaundice, cholangitis, cholangioscopy

## Abstract

Mucobilia is defined as the abnormal secretion of copious amounts of mucus from within the bile ducts, manifesting as obstructive jaundice or cholangitis. A spectrum of mucin-producing hepatobiliary and pancreatic neoplasms have been associated with mucobilia. We report the case of a 63-year-old male with an intestinal type of intraductal papillary neoplasm of the intrahepatic bile duct.

## Introduction

Mucobilia is a rare cause of biliary obstruction, with a reported incidence of less than 100 in cases of mucin-producing cholangiocarcinomas (MPCC) [1-3]. It can be found as an incidental finding on imaging or patients can present with symptoms attributable to biliary obstruction. Diagnosis can be established with imaging studies, such as ultrasound (USG), computerized tomography (CT) scan, or magnetic resonance imaging (MRI). Management includes the clearance of mucin. Cholangioscopy offers the benefit of biopsy. Surgery is the gold standard of treatment. Prognosis is good, with 5-year survival rates approximating 70%.

## Case presentation

**Figure 1:**
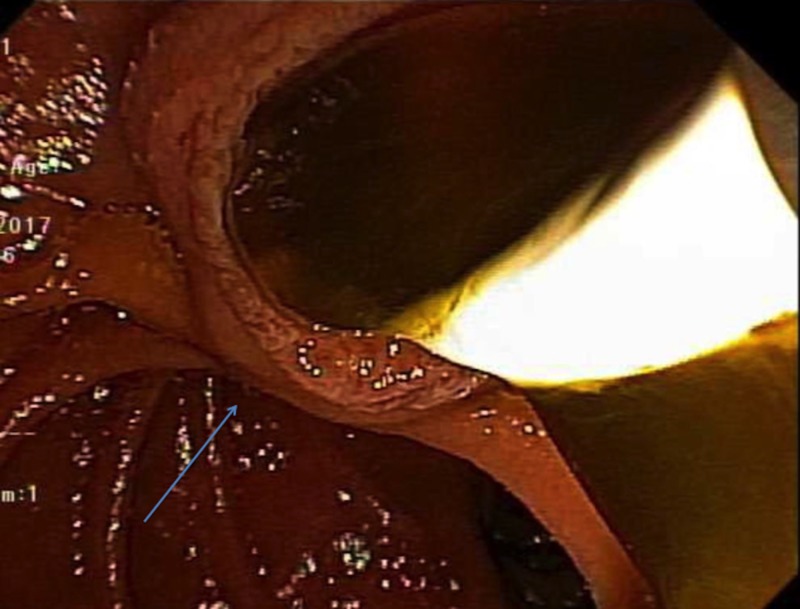
Endoscopic mucus retraction with balloon seen on ERCP ERCP: Endoscopic retrograde cholangiopancreatography

A 63-year-old caucasian male with hepatitis C presented with right upper quadrant pain, nausea, and dark urine. On physical examination, he was markedly jaundiced and tender to palpation in the right upper quadrant. Total bilirubin was elevated to 20.2 milligrams per deciliter (mg/dl) with a direct component of 15.0. Albumin was normal and International Normalized Ratio (INR) was 1.0. Aspartate aminotransferase (AST) and alanine aminotransferase (ALT) were 55 and 43 units per liter. Alkaline phosphatase (ALP) was 148 international units per liter. An ultrasound of the right upper quadrant of the abdomen showed a thickened gallbladder wall but no stones. A computed tomography (CT) scan of the abdomen was suggestive of early acute cholecystitis and common bile duct (CBD) dilation of 7 millimeters (mm). The patient was started on intravenous piperacillin/tazobactam 4 grams every six hours. An endoscopic retrograde cholangiopancreatography (ERCP) demonstrated dilated intrahepatic ducts and the occlusion of the CBD due to thick mucus and no pancreatic lesions (Figure 1).

**Figure 2:**
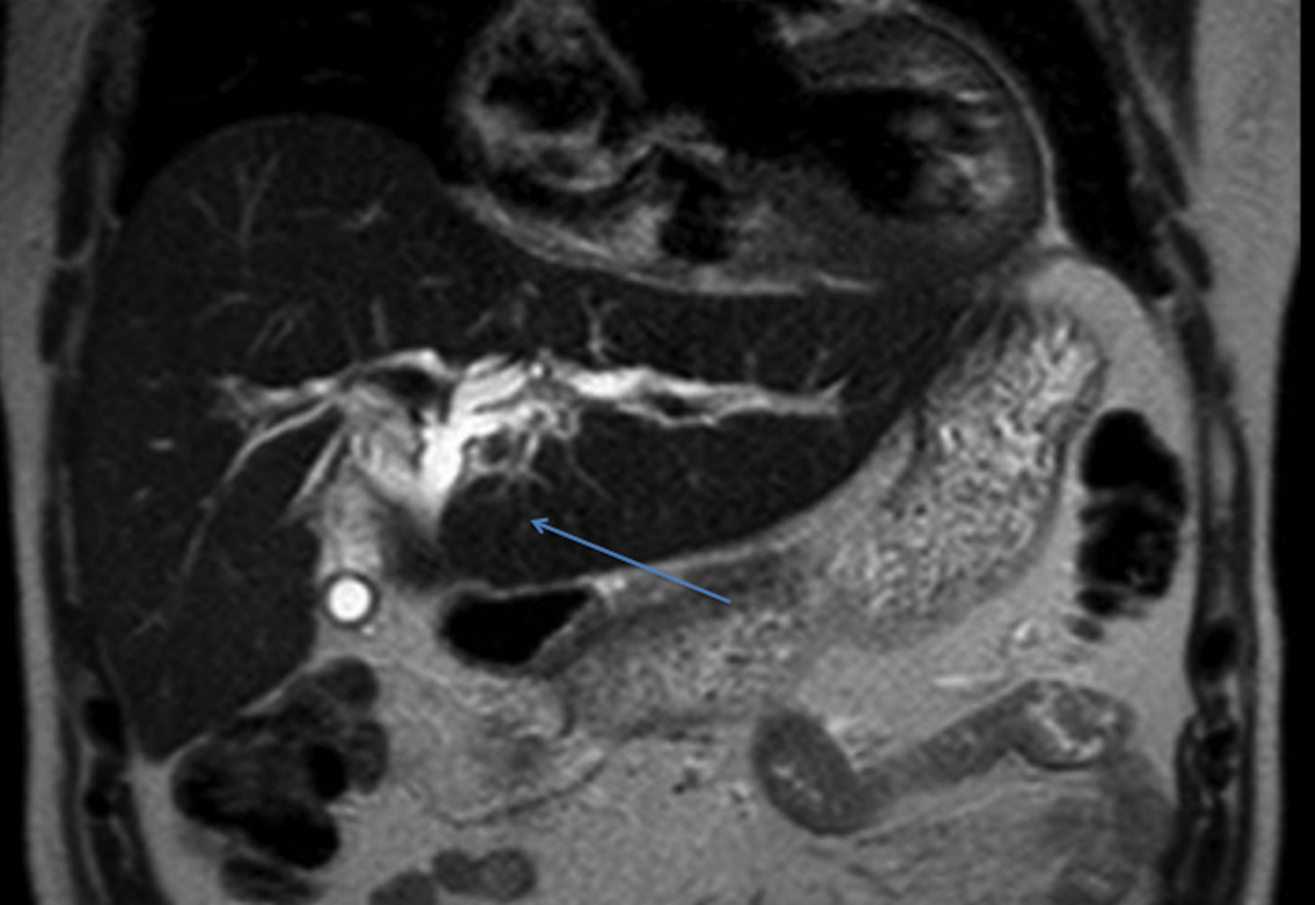
MRI of the abdomen demonstrating intrahepatic biliary dilation with no obvious mass MRI: Magnetic resonance imaging

A cholangiogram showed diffuse moderate dilation of the biliary tree and several filling defects in the CBD. The patient improved clinically with a rapid decrease in hyperbilirubinemia after clearing of the mucus and placement of a plastic biliary stent. Magnetic resonance imaging (MRI) of the abdomen showed intrahepatic biliary dilation but failed to reveal the cause of mucin production (Figure 2).

**Figure 3:**
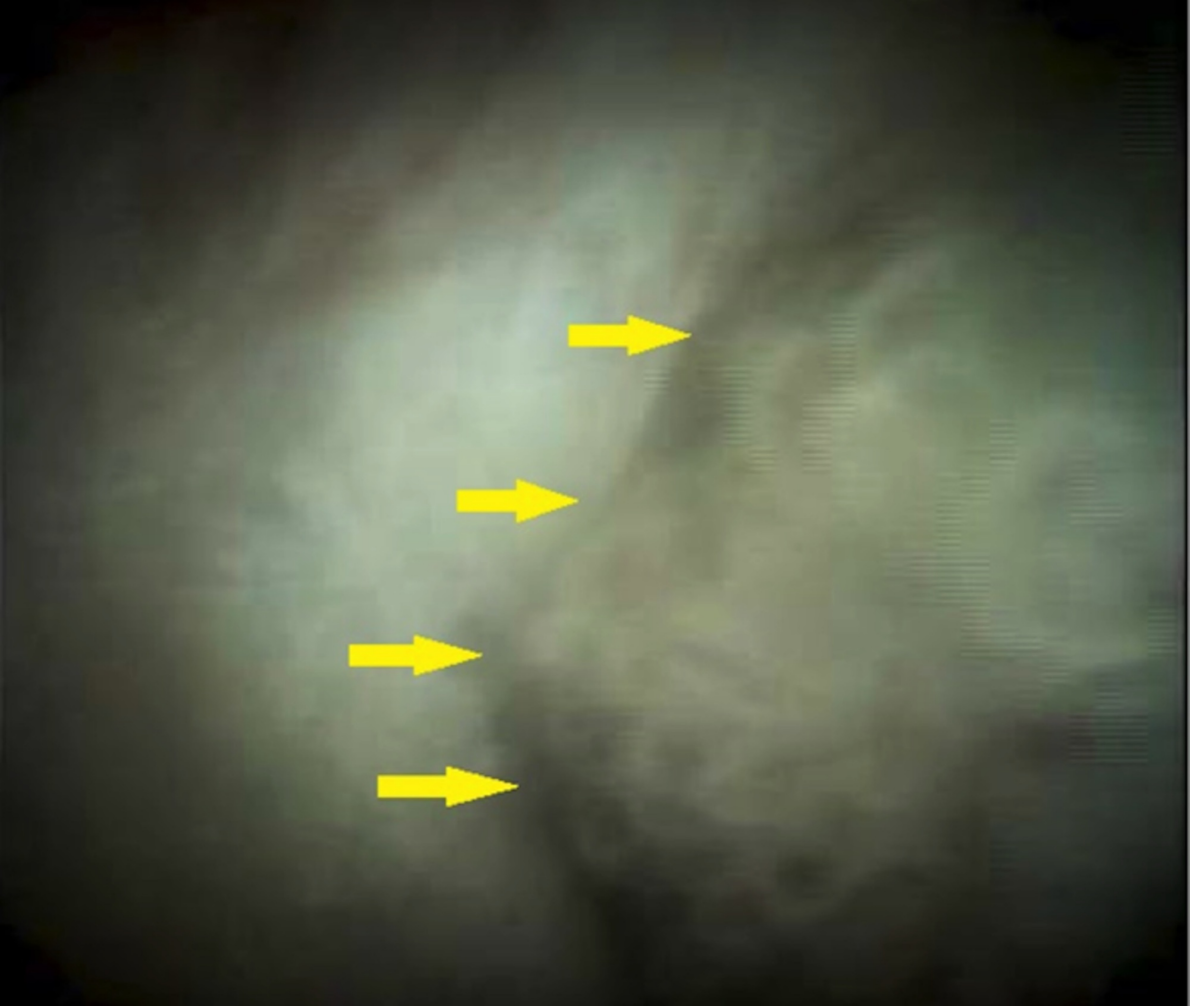
Cholangioscopy demonstrating a mucin-producing mass

He was shortly discharged thereafter for a referral for a repeat ERCP with digital cholangioscopy, which showed polypoid frond-like intraductal growth and associated mucin production, well within segment VIII of the intrahepatic biliary tree (Figure 3).

Cold forceps biopsies performed during cholangioscopy showed columnar mucosa with adenomatous changes. The patient underwent a laparoscopic converted to open right hepatectomy. Pathologic examination revealed an intraductal papillary neoplasm, intestinal type, with focal high-grade dysplasia. The patient did remarkably well and had normal liver function tests and an abdominal ultrasound with no evidence of recurrence at a six-month follow-up.

## Discussion

The differential diagnosis of mucobilia includes benign neoplasms, such as biliary mucinosis, biliary papillomatosis (BP), and cystadenomas of the pancreas or bile duct, and malignant neoplasms such as mucin-producing cholangiocarcinoma (MPCC) and cystadenocarcinomas of the pancreas, liver, or biliary tree. Tu et al. performed a retrospective analysis of 349 patients with obstructive jaundice, identifying mucobilia in 2.3% of these patients (8/349), the most common etiology being BP [1]. Kuo et al. reported mucobilia in 18% of 132 patients with MPCC [2]. In a retrospective review of 113 cases of biliary cystadenocarcinomas (BCCA) performed by Lauffer et al., they found 16 patients who had reported jaundice as the symptom [4].

Most of the signs and symptoms seen in a patient with mucobilia can be attributed to biliary obstruction. These patients can also present with right hypochondrial abdominal pain, fever with chills, and altered mental status, similar to a patient with ascending cholangitis [1-2]. An abdominal mass may often also be the presenting symptom. Bone pain or ascites, when present, are indicative of advanced disease [4-5]. Interestingly, biliary obstruction and jaundice rarely occur despite the presence of voluminous secretions.

Serologic tumor markers, including alpha-fetoprotein (AFP), carcinoembryonic antigen (CEA), and CA 19-9, can be elevated but are non-specific. Imaging studies, including ultrasound, computed tomography (CT) scan, or magnetic resonance imaging (MRI), have a high sensitivity but the specificity is not known [4,6]. Mucin has a similar signal intensity as that of bile and can be identified as hyperechoic spots within the dilated ducts [6]. Yang et al. demonstrated that the amount of mucin secretion has no association with the degree of the atypia of the tumor cells [7]. T2-weighted images can be helpful to differentiate hyperintense mucin from other intraluminal filling defects, such as stone, mass, or a blood clot, which typically appear as a signal void or as hypointense or isointense [8]. A hypodense cystic lesion with internal septa or papillary projections may provide a clue for the diagnosis. Biliary cystadenocarcinomas show internal nodularity and septation, compared to biliary cystadenomas that show septation without nodularity. Biliary cystadenomas and cystadenocarcinomas include a solitary cystic mass with a well-defined, thick, fibrous capsule. Other common findings encountered are mural nodules, internal septa, and, occasionally, capsular calcification. Endoscopic retrograde pancreatography (ERCP) or percutaneous transhepatic cholangiography (PTC) can also be used to demonstrate the presence of direct communication between the cyst and intrahepatic ducts and allow for therapeutic decompression of the biliary tree. Fine needle aspiration (FNA) is generally contraindicated due to the risk of dissemination of tumor cells.

The term intraductal papillary mucinous neoplasm (IPMN) was coined in the 1990s. They were established as a unique entity as part of the 2000 World Health Organization classification [9]. IPMN is an intraductal tumor whose papillary epithelial proliferation and mucin production cause cystic dilatation of the involved ducts. They can be morphologically defined as a grossly visible (>1.0 cm) intraductal epithelial neoplasm composed of mucin-producing columnar cells. These cells can demonstrate papillary proliferation, cyst formation, and variable degrees of cellular atypia. They can be classified based on their site of origin into the main duct and branch duct types. They can also be categorized based on their histological appearance into intestinal, pancreatobiliary, oncocytic, and gastric types. The gastric type is predominantly seen in the branch ducts while the former three are more commonly seen in the main duct. Although there are no well-established causative factors, the majority of these patients are former cigarette smokers.

The management of patients with mucobilia should include the clearance of as much intraductal mucin as possible. Endoscopic nasobiliary drainage (ENBD) can be employed for this purpose. Electrolyte abnormalities after external biliary drainage are not uncommon given the high concentration of albumin and electrolytes within the mucus and should be managed appropriately. Peroral cholangioscopy (POCS) has been proven to be a useful diagnostic tool in the evaluation of patients with mucobilia [10-12]. Tu et al. employed cholangioscopy for biopsy in a series of eight patients in 2003. The ultimate advantage of direct cholangioscopy, which makes it the preferred diagnostic tool in mucobilia, is that it permits the biopsy of biliary lesions. Hepatolithiasis, as a result of the mucin or the primary disease process itself, increases the risk of cholangiocarcinoma (CCA). Surgery remains the gold standard of treatment for all patients with malignant biliary obstruction, with or without mucobilia. Diagnostic laparoscopy is recommended in all patients, to rule out metastatic disease. Patients found to have a malignant process may require a partial hepatectomy with common bile duct (CBD) exploration.

In general, prognosis and reported survival rates for patients with papillary tumors of the bile duct are higher than those reported for patients with other hepatopancreatobiliary tumors. In a study by Lee et al., survival rates for non-mucin secreting BP were significantly higher as compared to mucin-secreting BP (MBP) at one, three, and five years (89% versus 69%, 57% versus 37%, and 52% versus 19%) [13]. Studies have reported the presence of mucin to be a good prognostic sign in cases of papillary malignancy, although its presence in benign conditions might confer a worse prognosis [14]. MPCC patients had a better prognosis than non-MPCC patients [2]. The overall five-year survival for patients with a BCCA undergoing surgical resection is 65% to 71%. If complete surgical resection with negative histologic margins is achieved, a two- and five-year survival rate of 100% and a recurrence rate of 13% have been reported [4,15].

## Conclusions

Mucobilia is associated with multiple benign and malignant mucin-producing pancreatic and biliary neoplasms. When present, it confers a favorable prognosis in case of malignant conditions. It can be encountered as an incidental finding or manifest clinically as jaundice. Imaging is key in establishing the etiology. Intrahepatic biliary ductal dilatation in a patient with jaundice should alert the clinician to consider this diagnosis. We propose that in addition to the usual workup for obstructive jaundice, evaluation of mucobilia should include cholangioscopy and endoscopic ultrasound (EUS). Cholangioscopy allows the direct visualization of intraductal mucin-producing neoplasms and permits the biopsy of biliary lesions, which makes it a preferred diagnostic modality. EUS helps in identifying any hepatobiliary and pancreatic lesions that may have been missed. Surgery remains the mainstay of therapy, with complete tumor resection with negative histologic margins resulting in a cure.
